# Lysosome Alterations in the Human Epithelial Cell Line HaCaT and Skin Specimens: Relevance to Psoriasis

**DOI:** 10.3390/ijms20092255

**Published:** 2019-05-07

**Authors:** Katarzyna Bocheńska, Marta Moskot, Marcelina Malinowska, Joanna Jakóbkiewicz-Banecka, Aneta Szczerkowska-Dobosz, Dorota Purzycka-Bohdan, Joanna Pleńkowska, Bartosz Słomiński, Magdalena Gabig-Cimińska

**Affiliations:** 1 Department of Medical Biology and Genetics, University of Gdańsk, Wita Stwosza 59, 80-308 Gdańsk, Poland; katarzyna.bochenska@phdstud.ug.edu.pl (K.B.); marta.moskot@biol.ug.edu.pl (M.M.); marcelina.malinowska@biol.ug.edu.pl (M.M.); joanna.jakobkiewicz-banecka@biol.ug.edu.pl (J.J.-B.); joanna.plenkowska@phdstud.ug.edu.pl (J.P.); 2 Institute of Biochemistry and Biophysics, Polish Academy of Sciences, Laboratory of Molecular Biology, Kładki 24, 80-822 Gdańsk, Poland; 3 Department of Dermatology, Venereology and Allergology, Medical University of Gdańsk, Mariana Smoluchowskiego 17, 80-214 Gdańsk, Poland; aneta.szczerkowska-dobosz@gumed.edu.pl (A.S.-D.); purzycka-bohdan@gumed.edu.pl (D.P.-B.); 4 Department of Immunology, Faculty of Medicine, Medical University of Gdańsk, Dębinki 1, 80-211 Gdańsk, Poland; bartosz.slominski@gumed.edu.pl

**Keywords:** skin disorder, psoriasis, in vitro and in situ studies, human cell line and skin specimens, lysosomal compartments

## Abstract

Despite the constantly updated knowledge regarding the alterations occurring in the cells of patients with psoriasis, the status and the role of the lysosome, a control center of cell metabolism, remain to be elucidated. The architecture of the epidermis is largely regulated by the action of lysosomes, possibly activating signaling pathways in the cellular crosstalk of keratinocytes—epidermal cells—with infiltrating immune cells. Thus, in the present study, lysosome alterations were examined in vitro and in situ using a two-dimensional (2D) keratinocyte model of HaCaT cells with “psoriasis-like” inflammation and skin specimens, respectively. Specific fluorescence and immunohistochemical staining showed an augmented level of acidic organelles in response to keratinocyte activation (mimicking a psoriatic condition while maintaining the membrane integrity of these structures) as compared with the control, similar to that seen in skin samples taken from patients. Interestingly, patients with the most pronounced PASI (Psoriasis Area and Severity Index), BSA (Body Surface Area), and DLQI (Dermatology Life Quality Index) scores suffered a high incidence of positive lysosomal-associated membrane protein 1 (LAMP1) expression. Moreover, it was found that the gene deregulation pattern was comparable in lesioned (PP) and non-lesioned (PN) patient-derived skin tissue, which may indicate that these alterations occur prior to the onset of the characteristic phenotype of the disease. Changes in the activity of genes encoding the microphthalmia family (MiT family) of transcription factors and mammalian target of rapamycin complex 1 (*MTORC1*) were also observed in the in vitro psoriasis model, indicating that the biogenesis pathway of this arm is inhibited. Interestingly, in contrast to the keratinocytes of HaCaT with “psoriasis-like” inflammation, *LAMP1* was up-regulated in both PP and PN skin, which can be a potential sign of an alternative mechanism of lysosome formation. Defining the molecular profile of psoriasis in the context of “the awesome lysosome” is not only interesting, but also desired; therefore, it is believed that this paper will serve to encourage other researchers to conduct further studies on this subject.

## 1. Introduction

In recent years, increasing attention has been paid to the contribution of lysosomes to autoimmune diseases [1,2]. Of all the cellular components, the lysosome is an obvious candidate to play a role in the inflammatory process [3]. The relatively recent, intensively developing field of “lysosomics” provides a range of important information regarding the role of lysosomes in the inflammatory response and autoimmune conditions [4,5,6,7], despite the fact that the first signs of lysosomal involvement in inflammatory diseases were identified decades ago [8]. Unfortunately, these investigations were only superficially concerned with inflammatory skin diseases, including the most common human skin autoimmune disease, psoriasis (PsO) [9,10,11,12]. PsO, considered to develop through the crosstalk between the epidermal keratinocytes and immunocytes, is a polygenic, chronic, inflammatory skin disease affecting approximately 2−3% of the population worldwide. It is characterized by keratinocyte hyperproliferation, abnormal epidermal differentiation, and infiltration of immune cells into lesions [13]. Research carried out in the 1960’s highlighted the differences in the presence of lysosomes in the keratinocytes of psoriatic skin in relation to healthy skin [14]. Several subsequent studies have shown that affected psoriatic skin differs from normal skin with respect to the level of activity of lysosomal hydrolytic enzymes and membrane proteins [15,16,17,18]. Nevertheless, the outcomes of these studies are generally contradictory, since both increases and decreases in the activity of these enzymes were observed [18]. It should be mentioned, however, that this phenomenon was determined in various tissues and cells such as skin, scales covering psoriatic papules, lymphocytes, granulocytes, neutrophils, and blood serum, which could have led to these discrepancies. The observations have become increasingly widespread, since multiple studies have reported on the manner by which disruption of normal lysosomal function leads to abnormalities in inflammation and immunity [1,6,18,19,20]. The link between lysosomes and skin inflammation was established by studies conducted in the 1970’s [21,22,23]. Inflammation in skin diseases, such as PsO, is regulated by a complex network of keratinocytes and immune cells that infiltrate the epidermis [24]. Understanding of the lysosome signaling in the immune response is still in its infancy, same as knowledge of the potential involvement of lysosomes and lysosomal compartments in the inflammatory signaling network in respect to inflammatory skin diseases is residual. The question is, how can we move forward to gain further information and a greater understanding of this issue. To carry out studies assessing the potential contribution of lysosomes to the disturbed processes of apoptosis and autophagy, abnormalities of sphingolipid and ceramide metabolism pathways, incorrect presentation of antigens by major histocompatibility complex (MHC) class I and II molecules or increased transcription of genes encoding selected proinflammatory cytokines and chemokines—processes being deregulated in this dermatosis, the gaps in knowledge must be filled using recently developed “lysosomics”, therein in relevance to psoriasis. Hence, in vitro and in situ study of the pattern of the lysosomal system, a potential target for therapeutic intervention in human autoimmune-mediated inflammatory skin diseases, was the focus of the present research report. In addition to the human immortalized keratinocyte HaCaT model, skin tissue from biopsies of controls, healthy individuals, and patients with psoriasis, from the involved and noninvolved psoriatic skin, were evaluated.

## 2. Results 

### 2.1. Alterations in the Extent of Lysosomal Compartments and Other Acidic Vesicles in Response to Keratinocyte Activation, Mimicking Psoriatic Condition 

**Figure 1:**
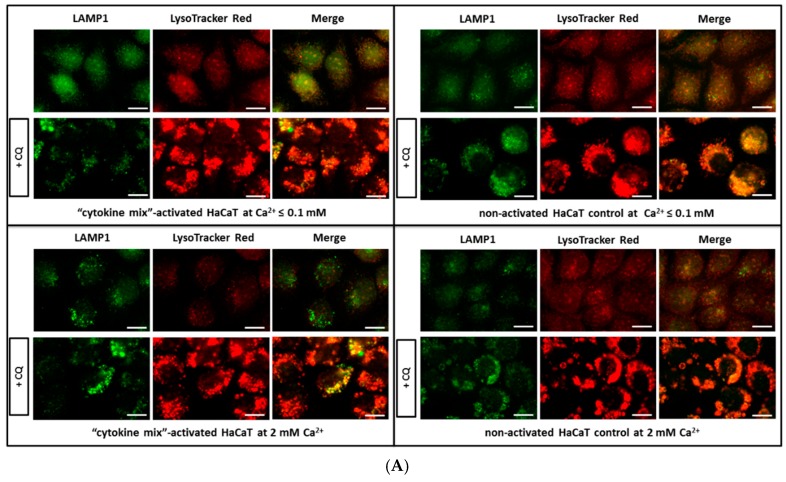

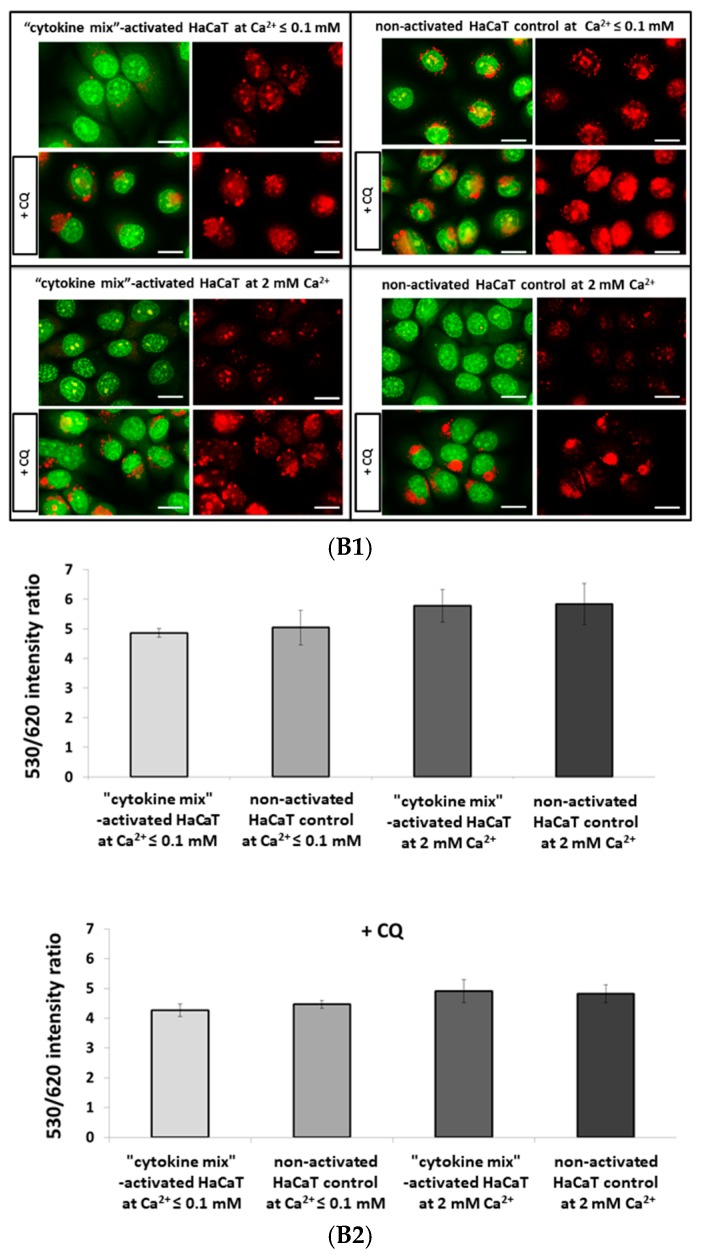
Fluorescence microscopy analysis of lysosomal compartments and other acidic vesicles in keratinocytes. Shown are representative microscopy images of HaCaT cells: (i) Activated with “cytokine mix”: interleukin 1 alpha (IL-1α), IL-17A, IL-22, oncostatin M (OSM), and tumor necrosis factor alpha (TNF-α) at a concentration of 2 ng/mL each constituent and cultured in medium containing Ca^2+^ ≤ 0.1 mM; (ii) non-activated HaCaT cell control cultured in medium containing Ca^2+^ ≤ 0.1 mM; (iii) “cytokine mix”-activated HaCaT cells cultured in medium containing 2 mM Ca^2+^; and (iv) non-activated HaCaT cell control cultured in medium containing 2 mM Ca^2+^. Control with chloroquine (CQ) was used to increase the total amount of lysosomes. All images were captured from randomly selected ten microscopic fields containing 50–100 cells, each from three independent experiments (*n* = 3), using a fluorescence microscope. Scale bar represents 0.25 µm. (**A**) LysoTracker Red DND-99 staining showing the distribution of acidic organelles, including mature lysosomes and lysosomal-associated membrane protein 1 (LAMP1) staining of the lysosomal membrane. (**B1**) Lysosomal membrane permeabilization detection and visualization of acidic vesicles (AVOs) loaded with acridine orange (AO). DNA and RNA are stained green by acridine orange and healthy lysosomes are stained red. Upon exposure to chloroquine (positive control for lysosomal membrane permeabilization), lysosomes increase in size and stain green and yellow, indicating lysosomal swelling. (**B2**) Acridine orange assay measuring fluorescence intensity emission at 530 and 620 nm following excitation at a wavelength of 490 nm. The 530/620 nm ratio indicates cytoplasm to acidic vesicle rate. Each treatment was performed in 20 wells. Data represent the mean values ± standard deviation (SD) from *n* = 3.

Firstly, quantitative assessment and distribution of acidic cell compartments, lysosomal membrane permeabilization, and maintenance of the intracellular (lysosomal–cytosolic) pH gradient in proinflammatory cytokine- and vehicle-treated HaCaT keratinocytes was performed. To maintain the cells in an undifferentiated state, thus imitating as much as possible a “psoriasis-like” inflammation status of HaCaT cells, the calcium ion concentration in the culture medium (Ca^2+^ lower than or equal to 0.1 mM) was decreased. Moreover, supplementary control assays were conducted by the addition of 2 mM calcium chloride to best mimic the differentiated keratinocyte phenotype, thus reflecting the normal state of skin health. The characteristics were evaluated by fluorescent microscopy using LysoTracker Red DND-99 (Figure 1A), an antibody against lysosomal-associated membrane protein 1 (anti-LAMP1) also known as lysosome-associated membrane glycoprotein 1 and CD107a (Cluster of Differentiation 107a) (Figure 1A), as well as with acridine orange (AO) (Figure 1B1,B2). Cells were divided into four groups from the same passage and cultured respectively as: (i) Keratinocytes of HaCaT with “psoriasis-like” inflammation with the addition of a combination of proinflammatory cytokines (“cytokine mix”): interleukin 1 alpha (IL-1α), interleukin 17A (IL-17A), interleukin 22 (IL-22), oncostatin M (OSM), and tumor necrosis factor alpha (TNF-α) (2 ng/mL each) in medium containing Ca^2+^ ≤0.1 mM; (ii) non-activated keratinocytes in medium containing Ca^2+^ ≤ 0.1 mM; (iii) keratinocytes of HaCaT with “psoriasis-like” inflammation with the addition of proinflammatory “cytokine mix” (as above) in medium containing 2 mM Ca^2+^; and (iv) non-activated keratinocytes in medium containing 2 mM Ca^2+^. Control with chloroquine (CQ) was used to increase the number of lysosomes in general (CQ blocks combining the lysosomes with autophagosomes). In general, the data clearly demonstrate that there was an increase in the quantity of lysosomes (LAMP1) with a reduction in the total amount of acidic vesicular structures (LysoTracker Red), while maintaining the membrane integrity of these structures (AO), in the keratinocytes of HaCaT with “psoriasis-like” inflammation as compared with the non-activated keratinocytes with a differentiated phenotype (2 mM Ca^2+^). A similar pattern is observed in the CQ control cell cultures for all conditions.

### 2.2. No Translocation of Endogenous Transcription factor EB (TFEB) to Nucleus in Keratinocyte Model of HaCaT Cells with “Psoriasis-Like” Inflammation

**Figure 2:**
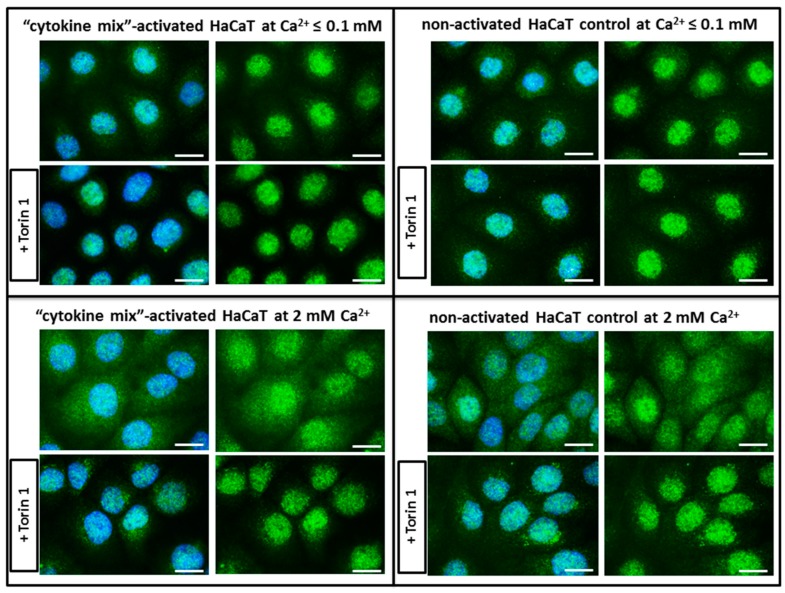
Immunofluorescence examination of endogenous transcription factor EB (TFEB) localization in: (i) HaCaT cells activated with “cytokine mix” and cultured in medium containing Ca^2+^ ≤ 0.1 mM; (ii) non-activated keratinocyte control cultured in medium containing Ca^2+^ ≤ 0.1 mM; (iii) “cytokine mix”-activated HaCaT cells cultured in medium containing 2 mM Ca^2+^; and (iv) non-activated keratinocyte control cultured in medium containing 2 mM Ca^2+^. Representative microscopy images from randomly selected ten microscopic fields containing 50–100 cells, each from three independent experiments (n = 3) of the tested keratinocyte cultures demonstrate the level of TFEB translocation from the cytoplasm to the nucleus. Scale bar represents 0.25 µm.

The results described above prompted examination of the alterations in the subcellular localization of endogenous TFEB, a master regulator of lysosome biogenesis and function. Among the acidic vesicles, the number of lysosomal compartments increased quantitatively in condition which mimics the psoriatic status, i.e., in response to keratinocyte activation. To test the potential role of TFEB-mediated lysosomal arrangement in the “psoriasis-like” inflammation phenotype of keratinocytes, TFEB cytoplasm-to-nucleus shuttling under proinflammatory cytokine-activated and non-activated conditions was evaluated in in vitro HaCaT cultures. Additional control assays were conducted with cells cultured in medium containing a high concentration of Ca^2+^ (2 mM). Interestingly, it was noted that in keratinocytes grown in medium containing a low concentration of calcium (Ca^2+^ ≤ 0.1), TFEB translocated from a diffuse cytoplasmic location to the nucleus, at a level comparable to the selective inhibitor of mammalian target of rapamycin (mTOR) kinase (Torin 1) positive control, regardless of whether the keratinocytes were activated psoriatically. Such an effect was not seen when the keratinocytes were cultured in medium containing a high concentration of calcium ions (Figure 2). Therefore, it was concluded that the calcium ions, rather than “cytokine-mix” stimulation, was responsible for this phenomenon.

### 2.3. Acidic Vesicles Ultrastructure Changes in Keratinocyte Model of HaCaT Cells with “Psoriasis-Like” Inflammation

**Figure 3:**
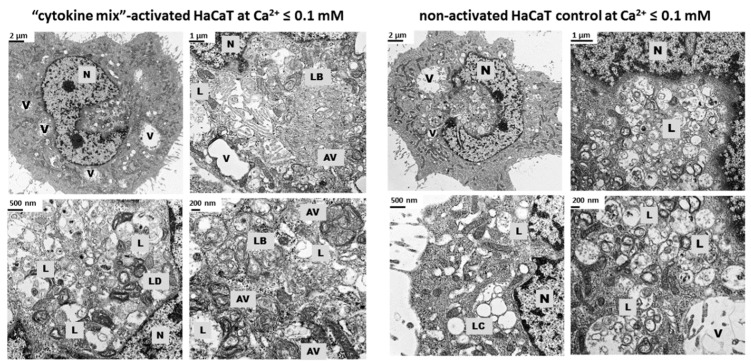
Transmission electron microscopy (TEM) micrographs showing the ultrastructure of HaCaT cells activated with proinflammatory cytokines (“cytokine mix”) and cultured in medium containing Ca^2+^ ≤ 0.1 mM vs. the non-activated keratinocyte control cultured in medium containing Ca^2+^ ≤ 0.1. Representative images were collected from at least 30 cells obtained from three independent cell cultures (*n* = 3). Scale bars are shown on each image. Abbreviations: N—nucleus; —autophagosomal vacuoles; L—lysosomes; LB—lamellar bodies; LC—carbohydrate-storing lysosomes; LD—lysosomes of amorphous flocculent and electron-dense structure; and V—vacuoles.

Intracellular structures, such as lysosomes and lysosome-related organelles (LROs), have a particular morphological signature that can only be appreciated by ultrastructural analysis at the electron microscopy level. The patterns undergo various modifications as a result of the action of various factors, including those linked to the occurrence of a specific disease. In the case of psoriasis, an in vitro model of keratinocytes that mimics the disease state by stimulation with a combination of proinflammatory factors in culture medium with a decreased calcium concentration was implemented. To accomplish the evaluation of the ultrastructure of both proinflammatory cytokine-treated and non-activated control HaCaT cells, transmission electron microscopy (TEM)-enabled visualization of representative acidic vesicle structures was performed. As shown in Figure 3, lysosomes (L), autophagosomal vacuoles (AV), lamellar bodies (LB), carbohydrate-storing lysosomes (LC), lysosomes of amorphous flocculent and electron dense structure (LD), and large vacuoles (V) could be depicted by TEM, which were predominant among the acidophilic organelles. In the control samples, the entire spectrum of autophagosomes and autolysosomes could be seen, whereas in the “psoriasis-like” sample, there were few autolysosomes and a dominance of autophagosomes with characteristic double-layered membranes in the form of multilamellar structures, somewhat similar to onion bodies, which were categorized as LROs. In addition, many more electron-lucent structures were seen in non-activated cells as compared with those activated with proinflammatory cytokines. Lastly, the keratinocytes in both experimental groups contained clusters of cellular compartments, mainly in the perinuclear area, as well as huge lysosome-like vacuoles (giant lysosomes) filled with small structures resembling mitochondria.

### 2.4. Increase of LAMP1 Expression in Psoriatic-Lesioned Skin

**Figure 4:**
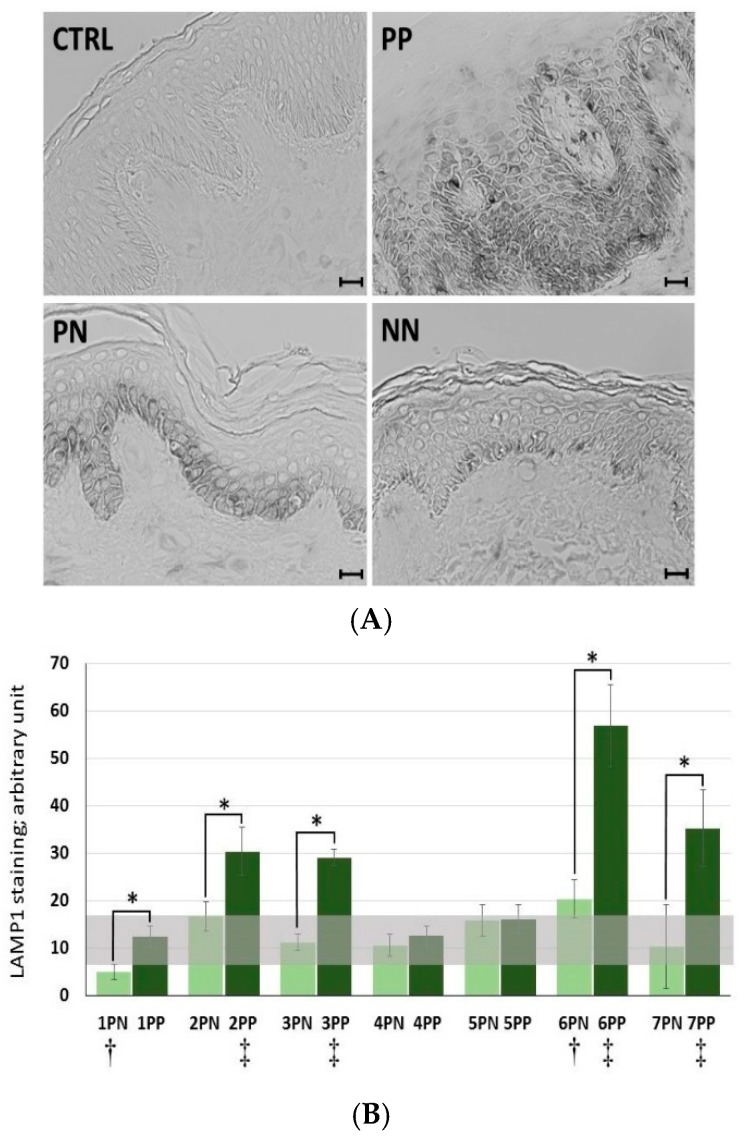
Immunohistochemical analysis of skin sections stained with an antibody against the lysosomal marker LAMP1 (lysosomal-associated membrane protein 1). (**A**) Representative images of LAMP1 staining of skin biopsies (black) from randomly selected six to eight microscopic fields, indicating the size and number of the lysosomal compartment. CTRL—IgG control for antibody specificity; PP—psoriatic plaque, PN—psoriatic normal, and NN—non-psoriatic normal. Scale bar represents 20 μm. (**B**) Quantitation of the mean LAMP1 staining in skin biopsies is expressed in arbitrary units. 6 to 8 low power fields of view for each sections were quantified. The horizontal lines show the 95^th^ percentile bootstrap confidence intervals. Comparisons among particular paired PP vs. PN, PP vs. mean NN, and PN vs. mean NN were performed, and statistical differences are observed following application of a nonparametric Mann-Whitney U test, with continuity correction (*p* ≤ 0.05). The asterisks designate a statistically significant difference between the PP and PN groups, while † and ‡ indicate a significant difference between the PN and NN, and PP and NN groups, respectively.

To confirm the alterations observed on the in vitro material, analyzes were performed also on the in vivo material, i.e., skin biopsies. Patients’ clinical data are summarized in Table 1. Immunohistochemical staining of LAMP1 in skin sections shows its expression in cells of the epidermis and dermis (Figure 4). It is worth noting that, irrespective of the sample type (PP—psoriatic plaque; PN—psoriatic normal; and NN—non-psoriatic normal), there were more LAMP1-positive cells in the epidermis as compared with the dermis (Figure 4A). In turn, in the epidermis, LAMP1 was mainly present in the basal layer; although, in the case of PP tissue sections from patients nos. 6 and 7, the LAMP1-positive cells were in the suprabasal layer, which is further in its spinous portion. In addition, it was rather simple to quantitate the LAMP1 signal in order to elucidate that the PP specimens had a greater number of LAMP1-positive cells as compared with those in the PN and NN skin sections, even though there were differences in the expression of this transmembrane glycoprotein among the skin samples from the tested individuals (Figure 4B). Comparison of the highest LAMP1 signal, obtained for PP and PN from patient no. 6, with the lowest, from patient no. 1, showed an increase of 4.6- and 4.1-fold, respectively (Table 2).

Immunohistochemical visualization of LAMP1 allowed the conclusion of a statistically significant increase in LAMP1 expression, and thus, a statistically important rise in the number of lysosomes in psoriatic lesions (PP) as compared with non-lesions (PN) in five (patients nos. 1, 2, 3, 6, and 7) among the seven tested patients. In addition, in four patients (nos. 2, 3, 6, and 7), there was a statistically significant greater number of lysosomes in PP as compared with NN (i.e., four individuals from the healthy control group), while in two patients (nos. 1 and 6), there was a statistically significant change (a reduction and an increase) in the number of lysosomes in PN as compared with NN.

Investigation into the association of the changes in LAMP1 expression in human skin specimens with clinical parameters revealed a subtle positive relationship in the examined patients (Table 1 and Table 2). Noteworthy, however, is the fact that patients nos. 6 and 7, characterized by high PASI (Psoriasis Area and Severity Index), BSA (Body Surface Area), and DLQI (Dermatology Life Quality Index) indices (for patient no. 6 with modest severity of disease: PASI 9.6, BSA 21%, and DLQI 19; and for patient no. 7 with mild severity of psoriasis: PASI 13.2, BSA 13%, and DLQI 16), had the highest LAMP1 expression in both the PP and PN samples among all those tested. Moreover, these two patients had the highest ratios (2.8, 5.4, and 1.9 for patient no. 6 and 3.4, 3.3, and 1.0 for patient no. 7, respectively) of PP vs. PN, PP vs. NN, and PN vs. NN among all those studied (Table 2). 

### 2.5. Deregulated Gene Expression Profile in Cellular and Biopsy Tissue Material

**Figure 5:**
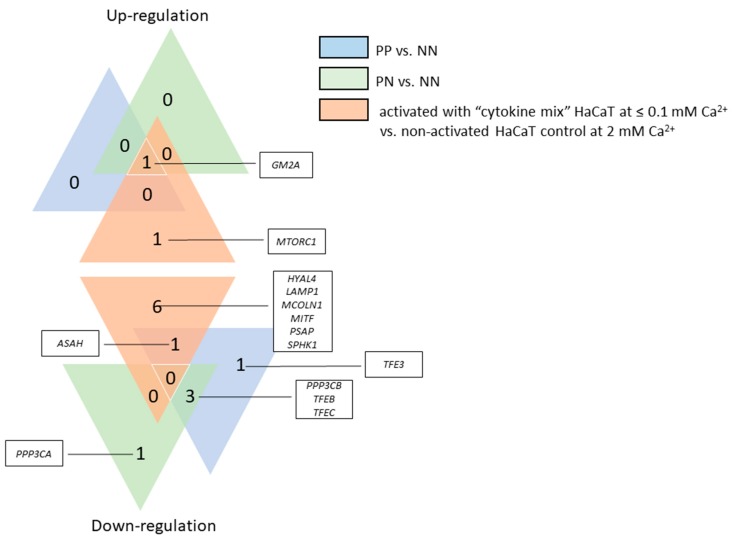
Genes with changes in expression identified via real-time qRT-PCR (0.7 ≥ FC ≥ 1.3) in the HaCaT cells with “psoriasis-like” inflammation and skin specimens of psoriasis patients 8 to 18, respectively, with the corresponding overlap between the datasets. The number in each triangle represents the amount of up- and down-regulated genes in the different comparisons: “cytokine mix”-activated HaCaT cells cultured in medium containing a Ca^2+^ concentration ≤0.1 mM vs. non-activated HaCaT cell control cultured in medium containing 2 mM Ca^2+^ (orange triangle); PP vs. NN skin (blue triangle); and PN vs. NN skin (green triangle). PP—psoriatic plaque; PN—psoriatic normal; NN—non-psoriatic normal (from healthy individuals 5 to 15).

For compilation of the data obtained from the in vitro model and the results of gene expression in situ (i.e., skin tissue), the expression profile of 17 genes, i.e., genes of the microphthalmia family (MiT family) of transcription factors (*MITF*, *TFE3*, *TFEB* and *TFEC*), lysosomal marker genes and those encoding factors that control the lysosomal biogenesis process (*LAMP1, MCOLN1, MTORC1, PPP3CA* and *PPP3CB*), and genes encoding lysosomal enzymes (*ASAH*, *GLA*, *GM2A*, *HPSE*, *HYAL4*, *PSAP*, *SMPD1* and *SPHK1*), were compared among the proinflammatory cytokine (“cytokine mix”) activated HaCaT cells cultured in medium containing a Ca^2+^ concentration ≤0.1 mM, the non-activated HaCaT cell control cultured in medium containing 2 mM Ca^2+^, and the PP vs. NN and PN vs. NN skin tissue from psoriasis patients (psoriasis patients 8 to 18) (Figure 5). Only the genes for which the expression was changed in all biological replicates were considered. The gene expression profile of the HaCaT cells with “psoriasis-like” inflammation in relation to skin tissue (both PP and PN) shows that the only common up-regulated gene (fold change, FC ≥ 1.3) was *GM2A*. In addition, increased expression of the *MTORC1* gene was seen only in activated HaCaT cells and in 73% of PP and 91% of PN skin samples (Figure S1). There were no common down-regulated genes in the HaCaT cells with “psoriasis-like” inflammation and skin specimens. Notably, the activity of six genes was decreased in HaCaT keratinocytes: *HYAL4*, *LAMP1*, *MCOLN1*, *MITF*, *PSAP*, and *SPHK1*. Comparison of the expression patterns between PP and PN skin samples showed a reduction in the activity of the *PPP3CB*, *TFEB*, and *TFEC* genes. Down-regulation of the *TFE3* gene was unique to PP skin samples, while decreased expression of the *PPP3CA* gene was characteristic of PN skin tissue.

#### 2.5.1. Modulated Transcript Level in the Human Epithelial Cell Model of HaCaT Keratinocytes with “Psoriasis-Like” Inflammation

Comparison of the human epithelial cell line HaCaT with “psoriasis-like” inflammation phenotype with control keratinocytes cultured in medium containing a low concentration of Ca^2+^ ions (≤0.1 mM) showed no significant changes in gene expression, with the exception of *HYAL4*, *PSAP* (down-regulated), and *SPHK1* (up-regulated). Keratinocytes cultured in medium containing a high concentration of Ca^2+^ ions (2 mM) mimics the normal differentiated keratinocyte phenotype, which is a more appropriate control for the “psoriasis-like” cell inflammation state (Table 3). Based on this comparison, decreased expression of the *MITF*, *TFEB*, and *TFEC* genes was observed in psoriatically activated keratinocytes, while the FC for the *TFE3* gene remained unchanged. Interestingly, expression of the TFEB inhibitory protein, *MTORC1*, was reduced, and the level of *MCOLN1* mRNA, which is involved in the opposing mechanism, i.e., nuclear translocation and activation of TFEB, was increased. This could result in the down-regulation of the *LAMP1* gene, although the level of genes encoding the calcineurin subunits (*PPP3CA* and *PPP3CB*) remained unchanged. The quantitative changes in lysosomes (reduction in the *LAMP1* marker) may result in the down-regulation of lysosomal enzymes or their cofactors. The exceptions were the *GLA*, *HPSE*, and *SMPD1* genes (unchanged, 1.3 ≥ FC ≥ 0.7) and *GM2A* (increased expression). Importantly, down-regulation of *IVL* expression (a marker for early keratinocyte differentiation) indicates that the cells remained immature, and up-regulation of the *S100A7* and *S100A9* genes proves that the inflammatory response was induced, which is characteristic of the psoriatic phenotype.

#### 2.5.2. Changes in Gene Activity in Human Skin Specimens

Gene expression studies included 17 genes in 11 psoriasis patients numbered from 8 to 18 (both in PP and PN skin tissue) and 11 healthy individuals numbered from 5 to 15 (NN skin tissue). All the tested genes were differentially expressed (0.7 ≥ FC ≥ 1.3). A summary of the values and percentage distribution pattern is listed in Table S1 and Figure S1.

**Figure 6:**
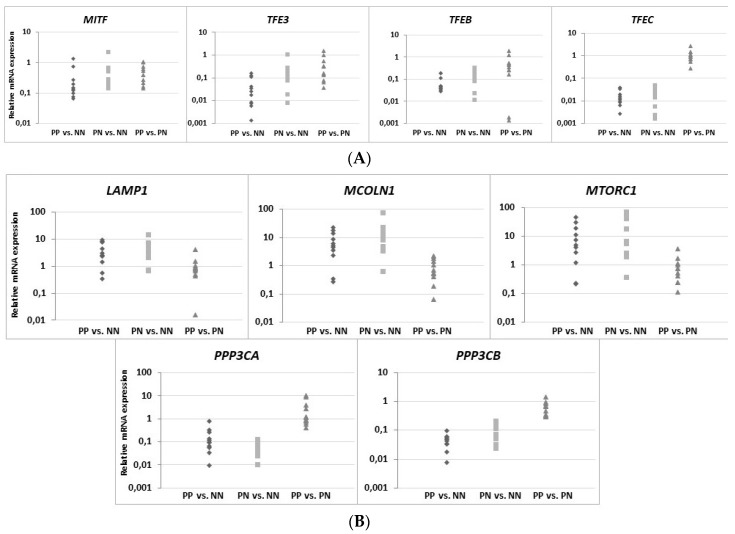

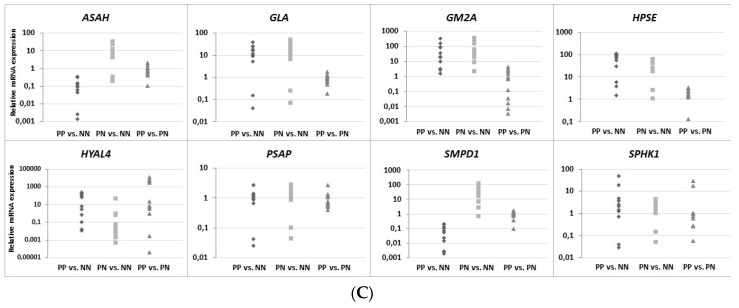
Real-time qRT-PCR verification of microphthalmia family (MiT family) genes (**A**: *MITF*, *TFE3*, *TFEB*, and *TFEC*); lysosomal marker genes and those encoding factors that control the lysosomal biogenesis process (**B**: *LAMP1*, *MCOLN1*, *MTORC1*, *PPP3CA*, and *PPP3CB*); and genes encoding lysosomal enzymes (**C**: *ASAH*, *GLA*, *GM2A*, *HPSE*, *HYAL4*, *PSAP*, *SMPD1*, and *SPHK1*) in skin specimens. The relative mRNA expression levels were determined against that of *GAPDH* (glyceraldehyde-3-phosphate dehydrogenase), and the relative quantitation was calculated using 2^−ΔΔ*C*t^ and compared between each group (PP—psoriatic plaque vs. NN—non-psoriatic normal, PN—psoriatic normal vs. NN, and PP vs. PN). Scatter plots showing the relative expression levels of genes, each with number of patients *n* = 11 (psoriasis patients 8 to 18) for PP/PN and *n* = 11 (healthy individuals 5 to 15 as a control) for NN.

The present study shows that the activity of the *TFE3*, *TFEB*, *TFEC*, *PPP3CB*, *ASAH*, and *SMPD1* genes was strongly decreased in psoriatic skin (PP) as compared with non-psoriatic normal skin (NN) in all patients regardless of clinical data (Figure 6), while the *GM2A* and *HPSE* genes were up-regulated in the same patients. Furthermore, a reduction in the activity of the *TFEB*, *TFEC*, *PPP3CA*, and *PPP3CB* genes and an increase in *GM2A* gene activity were observed in psoriatic normal skin (PN) as compared with non-psoriatic normal skin (NN) in all patients. Expression of the *MITF* gene was reduced in 82% of PP and 91% of PN skin specimens. Notably, the *ASAH* and *SMPD1* genes, encoding key enzymes for ceramide formation, were up-regulated in PN skin specimens (for 82% and 91% of patients, respectively), but down-regulated in PP skin specimens in all patients. The *SPHK1* mRNA level pattern was identical in PP and PN skin specimens, i.e., up-regulated in 64%, down-regulated in 18%, and unchanged in 18% of patients). Interestingly, the *GM2A* and *GLA* genes, encoding enzymes necessary for further conversion of ceramides, were increased in both PP and PN skin specimens, while expression of the *PSAP* gene was modulated differentially. The *HPSE* gene was overexpressed in 100% PP and 91% PN skin specimens, while up-regulation of the *HYAL4* gene was unique to PP skin tissue.

For the majority of patients (both PP and PN skin specimens), the *MTORC1* gene was overexpressed, while the *PPP3CA* and *PPP3CB* genes were down-regulated, which may result in decreased expression of genes belonging to the MiT family. Despite this, the *LAMP1* and *MCOLN1* genes had high FCs (in 82% of patients, including PP and PN skin), which may indicate that the number of lysosomes was increased but their biogenesis was independent of the MiT family. A comparable expression profile for PP vs. NN and PN vs. NN demonstrates that the above changes occur prior to the onset of the characteristic disease phenotype.

## 3. Discussion

The involvement and specific role of lysosomes and their components in the maintenance of cellular architecture is already well-documented in scientific reports on health and disease [4,25,26]; however, the potential impact of lysosomal signaling on autoimmune-mediated inflammation in skin diseases such as psoriasis (PsO) requires further verification. It appears that apart from human immune system cells, the epidermis—composed mostly of keratinocytes—may also be an important area of research, if only due to the proven interaction of both these cell types within a wide-range network regulating the biological pattern of the largest and fastest-growing organ of the human body—the skin.

Normal epidermal maturation is associated with an increased number of lysosomes involved in intracellular degradation. It is important to determine the potential consequences of changes in the amount and morphology of lysosomes and other acidic organelles in psoriasis, which may be manifested, among others, in disturbed processes of apoptosis and autophagy, abnormalities of sphingolipid and ceramide metabolism pathways, incorrect presentation of antigens by major histocompatibility complex (MHC) class II molecules or increased transcription of genes encoding selected proinflammatory cytokines and chemokines—processes being deregulated in this dermatosis. A significant number of alterations at the level of a single cell, affecting the homeostasis of the whole organism, can be largely explained by the characteristics of the action of lysosomes. They can play a key role in the modulation of the immune response, and thus may be a potential therapeutic target for many inflammatory disorders, such as rheumatoid arthritis, atopic dermatitis, and psoriasis. For this reason, in our study we undertook research on the verification of vicissitudes in the endosomal-lysosomal system, at this stage of work in cells mimicking psoriasis (keratinocyte model of HaCaT cells with “psoriasis-like” inflammation), with simultaneous analysis of material driven from people suffering from this dermatosis.

Despite the many controversies surrounding the use of proper epidermal keratinocytes for in vitro studies relevant to PsO, the most widely used human immortalized keratinocyte cell line, HaCaT, has been employed as a cellular model to investigate hyperproliferative skin diseases [27,28,29,30]. HaCaT keratinocytes are often used instead of primary keratinocytes (NHEK) for in vitro experiments related to psoriasis, since the susceptibility of primary cells to treatment may change with increasing numbers of passages, whereas HaCaT provides an almost unlimited supply of identical cells, ensuring high reproducibility [31]. On the other hand, it is necessary to add here that HaCaT keratinocytes are not perfect in this sense. They have a sufficient limitation as a cellular model of psoriasis due to their treatment with proinflammatory cytokines, which may not sufficiently increase proliferation rate of cells. Thus, the studies of psoriasis based on HaCaT cells as an experimental model shall take into account this important phenomenon, and if only possible, accompanied by additional tests, e.g. using tissues taken from patients. In turn, it is also worth adding that, cultured primary human keratinocytes are frequently employed for studies of psoriasis; however, interpretation of experimental data may be complicated by donor to donor variability, the relatively short culture lifetime, and variations between passages. Here, we investigated the use of HaCaT cells, a long-lived, spontaneously immortalized human keratinocyte line which is able to differentiate in vitro, as a suitable model to become undifferentiated cells under appropriate experimental conditions, mimicking the clinical psoriatic situation as closely as possible. Going forward, an important issue regarding psoriasis research is the involvement of in vivo experiments; thus, in addition to the HaCaT model with “psoriasis-like” inflammation, skin specimens of lesioned and non-lesioned tissue were examined in comparison with healthy control samples.

To verify the possible quantitative changes in organelles belonging to the lysosomal system among the tested keratinocytes, fluorescence microscopy was first applied. Indeed, the data demonstrates alterations in the cellular status at the level of lysosomal compartments and lysosome-related organelles (LROs) (Figure 1). In all staining tests, i.e., in the presence of LysoTracker, anti-lysosomal-associated membrane protein 1 (LAMP1) antibodies, and acridine orange (AO), an augmented level of lysosomes in HaCaT cells with “psoriasis-like” inflammation was found relative to the non-activated HaCaT cell control cultured in medium containing either Ca^2+^ ≤ 0.1 mM or 2 mM Ca^2+^. A similar tendency was noticed in the immunohistochemical study of skin samples taken from psoriasis patients (Figure 4).

In the case of more than 70% of the tested patients (five of seven subjects), there was a statistically significant increase in LAMP1 expression in psoriatic lesioned (PP) versus non-lesioned (PN) skin, as well as in four of these five patients when PN was compared with non-psoriatic normal (NN) (Figure 4). Interestingly, it was found that patients with the most pronounced PASI (Psoriasis Area and Severity Index), BSA (Body Surface Area), and DLQI (Dermatology Life Quality Index) scores (patients nos. 6 and 7) had a high incidence of positive LAMP1 expression, which indicates a level of correlation between particular clinical parameters and LAMP1 manifestation (Table 2). However, no significant association was defined between LAMP1 expression and other clinical characteristics; nevertheless, proper assessment was limited due to the small number of evaluated patients. These results were confirmed by real-time qRT-PCR analysis in skin specimens (Figure 6 and Table S1). Up-regulation of the *LAMP1* gene (fold change, FC ≥ 1.3) was observed in 82% of psoriatic patients, including PP and PN skin specimens. However, in contrast to our results obtained in skin sections and the data of *LAMP1* expression detected in HaCaT cells (Transcripts Per Million, TPM: 298.9) [32], there was down-regulation of *LAMP1* in our HaCaT model with “psoriasis-like” inflammation (Table 3). Since the presence of LAMP1 is noticeable in the same cells in culture, as evidenced by immunofluorescence, the effect on *LAMP1* mRNA expression observed in the present study suggests post-transcriptional control of LAMP1 production in HaCaT cells. A possible explanation for the discrepancy in the levels of LAMP1 protein and mRNA in our cultures of keratinocytes is the presence of a feedback loop that degrades the mRNA transcript following synthesis of a large amount of protein. Moreover, electron microscopy identified a substantially increased population of autophagosomes and a reduced population of autolysosomes in keratinocytes with “psoriasis-like” inflammation as compared with the control (Figure 3). There may be a mechanism that moderates or even blocks efficient fusion of numerous lysosomes with autophagosomes in proinflammatory cytokine-treated HaCaT cells, resulting in a reduction in the autolysosome population in these cells. On the other hand, it is known from the literature that lysosomes and lysosome-like compartments are involved in epidermal differentiation, the stage of cell maturation that is impaired in PsO [33,34,35,36]. The role of lysosomes in cell differentiation is supported by several studies performed in various cell types including keratinocytes [36]. In the normal human epidermis, lysosomes in keratinocytes, revealed by LAMP1 staining, were mainly observed in the granular layer, perhaps as lamellar granules involved in the keratinization and desquamation processes, whereas in cultured keratinocytes, lysosomes were abundant in subconfluent conditions. LAMP1 expression is elevated in normal proliferating keratinocytes, while it is significantly down-regulated upon entering the differentiation stage. Furthermore, maturing keratinocytes reveal deregulated lysosomal biogenesis and function, which in turn may be linked to the action of the microphthalmia family (MiT family) transcription factors. Recent studies have led to the identification of a lysosome signaling pathway and gene network that regulate cellular clearance and metabolism [25,26]. Using this approach, it was discovered that lysosome status is coordinated by the transcription factor EB (TFEB), a member of the MiT family, and its translocation from the cytosol to the nucleus [6]. Since TFEB plays a fundamental role in cell homeostasis and globally modulated lysosomal function, an investigation into the endogenous TFEB subcellular localization in our in vitro model was carried out (Figure 2). Surprisingly, rather than being due to proinflammatory cytokine stimulation, the cytosol-to-nucleus shuttling of TFEB was calcium-dependent.

It is worth mentioning that RNA expression profiling studies, carried out to analyze differentially expressed genes (DEGs), revealed a number of changes in the expression of genes specific to lysosomal function or other signaling pathways mediating the pathogenesis of PsO [12,37,38]. Numerous genes associated with lysosomes are termed coordinated lysosomal expression and regulation (CLEAR), the elements of which are recognized by the MiT family factors, promoting gene transcription. With the exception of TFEB, the MiT family of transcription factors include MITF, involved in melanogenesis; TFE3, which promotes the expression of genes downstream of transforming growth factor beta (TGFβ) signaling; and TFEC, which plays a role in cell survival, growth, and differentiation. With respect to lysosomal metabolism studies in this field, an interesting observation is the dysregulation of sphingolipid metabolism, in particular alterations in ceramides being a central hub of the sphingolipid pathway, in inflammatory skin diseases such as psoriasis and atopic dermatitis [39]. Sphingolipids, as bioactive molecules with a putative involvement in inflammation, play an important role in epidermal signaling [40]. A wide range of chemical structures of sphingolipids (giving various types of these compounds, i.e., ceramides, ceramide-1-phosphate, sphingomyelin, sphingosine-1-phosphate, or glycosphingolipids) is reflected in their function in the skin, specifically in proliferation signaling, survival and apoptotic processes, cell migration and adhesion, or angiogenesis. The highest percentage (almost 50%) of all sphingolipids in the epidermis are ceramides; however, this quantity is much lower in psoriatic plaque skin, which through its anti-apoptotic effect, contributes to the excessive proliferation of keratinocytes [40,41]. Furthermore, quantitative changes of sphingolipids can cause increased transepidermal water loss (TEWL) and epidermal barrier dysfunction. Moreover, there is altered sphingosine conversion in psoriatic patients’ skin, in which the level of the proinflammatory and prosurvival sphingosine-1-phosphate form is more common. In the present study, up-regulation of the *ASAH* and *SMPD1* genes (encoding enzymes necessary for the production of ceramide) was observed in PN skin specimens, while down-regulation was observed in PP specimens (Figure 6 and Figure S1), which may indicate quantitative disturbances (decreased amounts) in ceramides, resulting in a lack of differentiation and excessive proliferation of keratinocytes, suppression of the apoptotic process, and finally impairment of the skin barrier. Increased *SPHK1* activity in PP tissue sections may additionally result in excessive synthesis of sphingosine-1-phosphate, contributing to the production of tumor necrosis factor alpha (TNF-α), activation of the nuclear factor kappa light chain enhancer of activated B cells (NF-κB) pathway, or secretion of interleukin 8 (IL-8).

On the other hand, the function of the sphingosine form is calcium release from acidic stores, essential for TFEB translocation, which in turn is mediated by mucolipin-1 (MCOLN1) and calcineurin. This mechanism results in the biogenesis of lysosomes and the activation of autophagy processes, which are not observed in psoriatic epidermis [42,43,44]. The *MTORC1* gene was up-regulated in the majority of psoriasis patients in the present study, while the opposite genes, *PPP3CA* and *PPP3CB*, encoding calcineurin subunits, were down-regulated, which consequently led to reduced mRNA belonging to the MiT family. Interestingly, there was both an up-regulation of *LAMP1* gene activity and an elevated level of signaling protein in psoriatic skin tissue, which may indicate that the overall number of lysosomes is increased in PP skin, but their biogenesis process dependent on the MiT family transcription factors is inhibited. Therefore, endoplasmic reticulum (ER) stress may be a potential cause of the formation of lysosomes [34].

It must be mentioned that there are certain limitations faced by the present study. For instance, the use of a restricted number of samples may introduce bias to this study; hence, future investigations including larger sample sizes are necessary to confirm the results of the present exploration. A series of further in vitro and in vivo experiments are in progress. We anticipate that our research group will report related studies to illustrate the molecular mechanisms involved in the regulation of lysosome signaling, even considering it as a potential target for therapeutic intervention in serious inflammatory skin diseases such as psoriasis.

## 4. Materials and Methods

### 4.1. Cell Line, Culture Media, and Reagents

Spontaneously transformed keratinocytes from histologically normal skin (HaCaT cell line) were purchased from the CLS Cell Lines Service GmbH (Eppelheim, Germany) [45]. HaCaT cells from passage 38−42 were maintained in Dulbecco’s Modified Eagle’s Medium (DMEM, Gibco, Thermo Fisher Scientific, Waltham, MA, USA) supplemented with 10% fetal bovine serum (FBS, Gibco, Thermo Fisher Scientific) and 1% antibiotic/antimycotic solution (Gibco, Thermo Fisher Scientific) at 37 °C in a humidified atmosphere containing 5% carbon dioxide (CO_2_).

### 4.2. Two-Dimensional (2D) Engineered Skin Psoriatic Model

HaCaT cells were cultured to 80% confluence in 25-cm^2^ flasks containing DMEM supplemented with 10% FBS and 1% antibiotic/antimycotic solution. Subsequently, cells were enzymatically digested using 0.025% trypsin−0.01% EDTA and seeded on 6-well plates at a density of 4 × 10^5^ cells/well in defined serum-free keratinocyte medium (Keratinocyte-SFM, Gibco, Thermo Fisher Scientific) supplemented with bovine pituitary extract (BPE) and epidermal growth factor (EGF) (Gibco, Thermo Fisher Scientific). Following a 24-h incubation, the medium was changed to Keratinocyte-SFM without growth supplements for a further 16 h. Cells were then stimulated with a combination of a proinflammatory cytokines (“cytokine mix”): interleukin 1 alpha (IL-1α), interleukin 17A (IL-17A), interleukin 22 (IL-22), oncostatin M (OSM), and tumor necrosis factor-α (TNF-α) (Gibco, Thermo Fisher Scientific) at a concentration of 2 ng/mL each constituent. For better evaluation of the results, two control conditions were carried out: (1) Untreated cells cultured in medium with a low concentration of calcium ions (≤0.1 mM) (Keratinocyte-SFM, Gibco, Thermo Fisher Scientific); and, (2) untreated cells cultured in medium with a high concentration of calcium ions (addition of 2 mM calcium chloride for 24 h) (Keratinocyte-SFM, Gibco, Thermo Fisher Scientific).

### 4.3. Ethical Consent and Study Design Regarding Skin Biopsies

The in vivo material, i.e., the skin tissue, was collected from 18 psoriasis patients (numbered from 1 to 18), i.e.,: 7 subjects (numbered from 1 to 7) for the immunohistochemistry studies and 11 subjects (numbered from 8 to 18) for the gene expression analysis; and 15 healthy individuals as a control (numbered from 1 to 15), i.e., 4 subjects (numbered from 1 to 4) for the immunohistochemistry studies and 11 subjects (numbered from 5 to 15) for the gene expression analysis, and the study was assembled within the framework of our collaboration with the Department of Dermatology, Venerology, and Allergology at the Medical University of Gdańsk. Signed informed consent was obtained from all subjects under protocols approved by the Independent BioEthics Committee of the Medical University of Gdańsk (NKBBN/161/2017 and NKBBN/161-634/2018). Patient data were classified with respect to gender, age, family history, BMI, and disease indicators such as PASI (Psoriasis Area and Severity Index), BSA (Body Surface Area), and DLQI (Dermatology Life Quality Index).

### 4.4. Determination of the Localization and Quantitative Changes in the Lysosomal Organelles via Fluorescence Microscopy

To visualize the acidophilic cell compartments, LAMP1 and acridine orange staining were used. Lysosomal membrane integrity and maintenance of the lysosomal−cytosolic pH gradient was assessed using acridine orange (AO) relocation techniques.

An equal number of HaCaT cells (1 × 10^5^ cells/well) was seeded on glass slides (Millicell EZ SLIDE 8-well glass, Millipore, Burlington, MA, USA) in supplemented DMEM (Gibco, Thermo Fisher Scientific) and cultured for 24 h. The culture medium was replaced with Keratinocyte-SFM (Gibco, Thermo Fisher Scientific) supplemented with BPE and EGF (Gibco, Thermo Fisher Scientific). Following a 6-h incubation, cells were cultured in Keratinocyte-SFM without growth supplements for a further 16 h. For subsequent experiments, cells were stimulated for 24 h with a combination of proinflammatory “cytokine mix”: IL-1α, IL-17A, IL-22, OSM, and TNF-α (Gibco, Thermo Fisher Scientific) at a concentration of 2 ng/mL each constituent. The control cells were left unstimulated in Keratinocyte-SFM containing a low concentration of calcium ions (≤0.1 mM) (Gibco, Thermo Fisher Scientific). An additional control without the cytokine mixture but with 2 mM Ca^2+^ was applied to simulate conditions prone to keratinocyte differentiation.

Images were captured immediately using a Leica DMI4000 B microscope (Leica, Wetzlar, Germany) and the LAS X software (ver 3.0.16120.2, Leica, Wetzlar, Germany) at 100× magnification, from randomly selected ten microscopic fields containing 50–100 cells, each from three independent experiments (*n* = 3).

#### 4.4.1. LAMP1 Detection Using a Specific Fluorescently Labelled Antibody

To assess the lysosomal number and distribution via LAMP1 staining, proinflammatory cytokine-stimulated HaCaT cells were fixed with 4% paraformaldehyde (PFA) for 10 min at room temperature (RT) and subsequently permeabilized with 0.1% Triton X-100 for 15 min at RT. Cells were then blocked with 3% BSA in PBS + 0.1% Tween for 1 h at 4 °C. Immunostaining was performed by overnight incubation with a LAMP1 primary antibody (#9091, Cell Signaling, Leiden, Netherlands; 1:200) in blocking solution at 4 °C. The following day, cells were washed three times with PBS and incubated with a secondary antibody (anti-rabbit IgG (H + L), F(ab′)_2_ fragment, Alexa Fluor^®^ 594 Conjugate; #8889, Cell Signaling, Leiden, Netherlands; 1:1000) in blocking solution for 1 h at 4 °C. After three washes with PBS, coverslips were mounted in SlowFade™ Diamond Antifade Mountant with DAPI (Invitrogen, Thermo Fisher Scientific). Each treatment was analyzed in triplicate in three independent experiments.

#### 4.4.2. Visualization of Acidic Organelles Using Acridine Orange (AO) Staining

Since AO is a metachromatic fluorophore that emits red fluorescence at high acidic pH (intact lysosome) and green fluorescence at low acidic pH (cytosol and nucleus), lysosomal membrane destabilization was monitored as a decrease in granular red fluorescence or an increase in diffuse cytoplasmic green fluorescence. Chloroquine (CQ), which raises lysosomal pH, was used as a positive control. Granular red puncta were also indicators of lysosome number.

HaCaT cells cultured on glass slides were treated with 50 µM chloroquine sulphate (Sigma-Aldrich, St. Louis, MO, USA) for 24 h in conjunction with proinflammatory cytokine stimulation. After three washes with PBS, cells were incubated with 50 nM AO in Keratinocyte-SFM (Gibco, Thermo Fisher Scientific) without growth supplements, for 15 min at 37 °C, followed by three further PBS washes. Each treatment was performed in triplicate, with and without CQ, in three independent experiments.

#### 4.4.3. Quantitation of Acidic Organelles Using AO Staining

AO can be used to stain and count acidic organelles such as endosomes, lysosomes, and autolysosomes. Upon blue light excitation (485 nm) at low pH (inside organelles), it emits orange fluorescence (peak at 620 nm) and green fluorescence (peak at 530 nm) in the cytoplasm. A ratio of 530/620 was adopted to estimate the cytoplasmic to lysosomal area in proinflammatory cytokine-stimulated HaCaT cells.

A total of 5 × 10^5^/well keratinocytes were seeded on 96-well plates containing supplemented DMEM (Gibco, Thermo Fisher Scientific) and cultured for 24 h. Subsequently, the culture medium was replaced with Keratinocyte-SFM (Gibco, Thermo Fisher Scientific) supplemented with BPE and EGF (Gibco, Thermo Fisher Scientific). Following a 6-h incubation, cells were cultured in Keratinocyte-SFM without growth supplements for a further 16 h. For subsequent experiments, cells were stimulated for 24 h with a combination of proinflammatory “cytokine mix”: IL-1α, IL-17A, IL-22, OSM, and TNF-α (Gibco, Thermo Fisher Scientific) at a concentration of 2 ng/mL each constituent. The control cells were left unstimulated in Keratinocyte-SFM containing a low concentration of calcium ions (≤0.1 mM) (Gibco, Thermo Fisher Scientific). An additional control without the cytokine mix but with 2 mM Ca^2+^ was applied to simulate conditions prone to keratinocyte differentiation.

After treatment, cells were washed three times with PBS and incubated with 50 nM AO in Keratinocyte-SFM (Gibco, Thermo Fisher Scientific) without growth supplements for 15 min at 37 °C, followed by three further washes with PBS. Fluorescence quantitation was performed using an EnSpire Multimode Fluorescence Plate Reader (Perkin Elmer, Waltham, MA, USA). Each treatment was performed in 20 wells in three independent experiments.

#### 4.4.4. Determination of the Subcellular Localization of Transcription Factor EB (TFEB)

To analyze the proinflammatory effect on TFEB nuclear translocation, cytokine-stimulated HaCaT cells were fixed with 4% PFA for 10 min at RT. For endogenous TFEB staining, cells were permeabilized with 0.1% Triton X-100 for 15 min at RT, followed by blocking with 3% BSA in PBS + 0.1% Tween for 1 h at 4 °C. Immunostaining was performed by overnight incubation with a TFEB primary antibody (#4240, Cell Signaling, Leiden, Netherlands; 1:1000) in blocking solution at 4 °C. The following day, cells were washed three times with PBS, and incubated for 1 h with a goat anti-rabbit IgG Alexa Fluor 488 secondary antibody (#A-11034, Invitrogen, Thermo Fisher Scientific,; 1:500) in blocking solution at 4 °C. Following an additional three washes with PBS, coverslips were mounted in SlowFade™ Diamond Antifade Mountant with DAPI (Invitrogen, Thermo Fisher Scientific). Each treatment was analyzed in triplicate in three independent experiments. An additional control with 0.3 µM Torin 1 (MTORC1 inhibitor) was applied for the last 3 h of treatment.

### 4.5. Lysosomal Ultrastructure Determination Using Transmission Electron Microscopy (TEM)

To determine the effect of proinflammatory stimulation on HaCaT cellular structures, 1.5 x 10^5^ cells/well were seeded on 12-well plates and cultured overnight. The culture medium was then replaced with Keratinocyte-SFM (Gibco, Thermo Fisher Scientific) supplemented with BPE and EGF (Gibco, Thermo Fisher Scientific). Following a 6-h incubation, cells were cultured in Keratinocyte-SFM without growth supplements for a further 16 h. For subsequent experiments, cells were stimulated for 24 h with a combination of proinflammatory “cytokine mix”: IL-1α, IL-17A, IL-22, OSM, and TNF-α (Gibco, Thermo Fisher Scientific) at a concentration of 2 ng/mL each constituent. The control cells were left unstimulated. Following treatment, cells were washed three times with PBS, fixed with 2.5% glutaraldehyde and subsequently with 1% osmium tetroxide and 1% potassium hexacyanoferrate (III), and dehydrated in ethanol. Ultrasections of Epon 812 resin (Sigma-Aldrich Produktions GmbH, Fluka, Steinheim, Germany)-embedded cells were stained with lead citrate and uranyl acetate and examined using a transmission electron microscope (CM100, Philips, Amsterdam, Netherlands). All photographs were taken using the iTEM program (Olympus Soft Imaging Solution, Tokyo, Japan). The keratinocytes had an intact membrane and visible cells structures (nucleus, mitochondria, and lysosomes). At least 30 cells obtained from three independent cell cultures (*n* = 3) were examined, and representative electron micrographs are shown (1650× magnification).

### 4.6. LAMP1 Evaluation Using Immunohistochemistry

Skin biopsies with a minimum diameter of 3 mm (two clippings in the study group of 7 subjects (numbered from 1 to 7): From the psoriatic plaque and the non-lesioned skin; and one clipping in the control group of 4 healthy individuals (numbered from 1 to 4)) were taken from the buttocks, fixed with 4% PFA in PBS, and embedded in paraffin. Subsequently, 5 µm-thick sections were mounted on Superfrost adhesive slides (Fisher Scientific, Hampton, NH, USA), and the epitopes were exposed by boiling in a 10-mM citric acid solution (pH 6.0). Endogenous peroxidase activity was inhibited using 3% hydrogen peroxidase in methanol for 5 min. Sections were blocked with 10% goat serum/1% BSA in PBS for 1 h and stained with mouse anti-LAMP1 (eBioH4A3, Invitrogen, Thermo Fisher Scientific; 5 µg/mL) in 1% BSA for 1 h at RT. Sections were subsequently washed in PBS and incubated with a biotinylated goat anti-mouse IgG secondary antibody (Vector Laboratories Inc, Burlingame, CA, USA; 4 µg/mL) in 10% normal goat serum (Vector Laboratories Inc)/1% BSA in PBS for 45 min at RT. Finally, an anti-biotin tertiary antibody (VECTASTAIN Elite ABC-Peroxidase Kit, Vector Laboratories Inc) was added for 30 min according to the manufacturer’s instructions. The reaction was developed using DAB substrate (Vector Laboratories Inc) according to the manufacturer’s instructions. For quantification analysis, nickel was added to the DAB substrate to obtain black staining. A non-specific mouse IgG was used as a control in place of the primary antibody. All comparative sections were performed at the same time under identical conditions. Images were taken using an IX51 Olympus inverted light microscope (Olympus, Tokyo, Japan) with a color CCD camera and the cellSens imaging software (ver 1.12, Olympus). Comparative images were taken under the same light conditions and processed in the same way.

Four to ten non-overlapping low power (20× magnification) fields of view were digitally photographed from each section using an IX51 Olympus inverted light microscope (Olympus) with a color CCD camera and the cellSens imaging software (ver 1.12, Olympus). Identical exposure settings were used for each stain, and all photographs were taken during one session. Images were transformed to eight-bit grey resolution and stored in a TIFF format. The ImageJ software (ver.1.8.0_112, National Institutes of Health, Bethesda, MD, USA) was used for quantitation of LAMP1 staining. The entire non-manipulated field of view was blinded and quantitated for each stain, whilst an unstained area was used to determine (and subtract) background staining for each section. An average optical density for each section and each patient was calculated, which were subsequently averaged to give a group mean for the sample for each patient. Statistical analyses of LAMP1 expression in skin biopsies was made by comparisons among particular paired PP vs. PN, PP vs. mean NN, and PN vs. mean NN, and statistical differences are observed following application of a nonparametric Mann–Whitney *U* test, with continuity correction (*p* ≤ 0.05).

### 4.7. RNA Processing

#### 4.7.1. Cell Cultures

Total RNA was extracted from cells using the High Pure RNA Isolation Kit (Roche Applied Science, Penzberg, Germany) following the manufacturer’s instructions. The quantity of each RNA sample was evaluated using Quant-iT™ RiboGreen™ RNA Assay Kit (Invitrogen, Thermo Fisher Scientific). In addition, the quality of each RNA sample was assessed using the RNA 6000 Nano Assay on the Agilent 2100 Bioanalyzer (Agilent Technologies, Santa Clara, CA, USA). The synthesis of cDNA from an RNA template was conducted using Transcriptor First-Strand cDNA Synthesis Kit (Roche Applied Science).

#### 4.7.2. Skin Specimens

Total RNA was extracted from skin biopsies of 11 psoriasis patients (numbered from 8 to 18) and 11 control group subjects (numbered from 5 to 15) with the RNeasy Mini Kit (Qiagen, Hilden, Germany) following the manufacturer’s instructions. The quality and quantity of each RNA sample was evaluated using the RNA 6000 Nano Assay on the Agilent 2100 Bioanalyzer (Agilent Technologies) and Quant-iT™ RiboGreen™ RNA Assay Kit (Invitrogen, Thermo Fisher Scientific). The synthesis of cDNA from an RNA template was conducted by Transcriptor First-Strand cDNA Synthesis Kit (Roche Applied Science).

### 4.8. Real-Time Quantitative Reverse Transcription PCR Assays for mRNA Analysis (Real-Time qRT-PCR).

Real-time quantitative reverse transcription polymerase chain reaction (real-time qRT-PCR) was carried out with the Single Tube Custom TaqMan Gene Expression Assays (Thermo Fisher Scientific) and the LightCycler1 480 Probes Master (Roche Applied Science) using the Light Cycler 480 II detection system (Roche Applied Science). Expression values were normalized against control genes: *GAPDH* and *TBP*. The TaqMan probes used were as follows: *ASAH* (Hs00602774_m1), *GAPDH* (Hs02786624_g1), *GLA* (Hs00609238_m1), *GM2A* (Hs00166197_m1), *HPSE* (Hs00935036_m1), *HYAL4* (Hs00202177_m1), *IVL* (Hs00846307_s1), *LAMP1* (Hs00931461_m1), *MCOLN1* (Hs01100653_m1), *MITF* (Hs01117294_m1), *MTORC1* (Hs00234508_m1), *PPP3CA* (Hs00174223_m1), *PPP3CB* (Hs00236113_m1), *PSAP* (Hs01551096_m1), *S100A7* (Hs01923188_u1), *S100A9* (Hs00610058_m1), *SMPD1* (Hs03679346_g1), *SPHK1* (Hs00184211_m1), *TBP* (Hs00427620_m1), *TFE3* (Hs00232406_m1), *TFEB* (Hs00292981_m1), and *TFEC* (Hs00992838_m1). Each experiment of real-time qRT-PCR analysis was repeated at least three times (n). In case of HaCaT cells they were biological replications and data are reported as the mean SD with *p* < 0.05 considered statistically significant. For skin specimens technical repetitions were conducted.

## 5. Conclusions

As a result of our work, we observed an increased number of lysosomes both in the keratinocytes with “psoriasis-like” inflammation and in 70% of patients (their lesioned and non-lesioned psoriatic skin compared to normal tissue). Quantitative changes of lysosomes may result in the deregulation of selected enzymes, e.g. those involved in the ceramide conversion pathway, and thus lack of differentiation and excessive proliferation of keratinocytes, inhibition of apoptosis and disturbed skin barrier. Increased *SPHK1* gene activity in psoriatic lesioned (PP) skin may additionally generate excessive synthesis of sphingosine phosphate 1, resulting in the production of tumor necrosis factor alpha (TNF-α), activation of the nuclear factor kappa light chain enhancer of activated B cells (NF-κB) pathway or secretion of interleukin 8 (IL-8).

For most patients (both PP and non-lesioned (PN) skin) we observed upregulation of *MTORC1* (transcription factor EB (TFEB) inhibitor) gene expression and downregulation of genes encoding calcineurin subunits *PPP3CA* and *PPP3CB* (TFEB activator), which results in decreased activity of genes encoding the microphthalmia family (MiT family) transcription factors (being responsible for lysosomes’ biogenesis). Despite this, there is an increased expression of the *MCOLN* and *LAMP1* genes, which may indicate: (1) An increased number of lysosomes; and (2) inhibition of the lysosomal biogenesis process dependent on the MiT family transcription factors. Lysosomes can arise from endoplasmic reticulum stress [34]. A similar expression profile for PP and PN vs. NN group may indicate that, the above changes occur before the appearance of the characteristic phenotype of the disease.

## Figures and Tables

**Table 1:** Patients’ clinical data including the PASI (Psoriasis Area and Severity Index), BSA (Body Surface Area), DLQI (Dermatology Life Quality Index), size of psoriatic plaque, thickness of psoriatic plaque, type of psoriasis, pruritus, arthralgia, psoriatic arthritis, treatment regimes, comorbidities, gender, and age.

Patient No.	PASI (0–72)	BSA (0–100%)	DLQI (0–30)	Size of Psoriatic Plaque (0: small <3 cm, 1: big >3 cm)	Thickness of Psoriatic Plaque (0: thin <0.75 mm, 1: thick >0.75 mm)	Type of Psoriasis (1: <40 yrs.; 2: >40 yrs.)	Pruritus (0: no, 1: mild, 2: modest, 3: severe)	Arthralgia (0: no, 1: yes)	Psoriatic Arthritis (0: no,1: yes)	Treatment Regimes	Comorbidities	Gender/ Age
**1**	3.3	15	5	1	1	2	2	1	0	Topical treatment	-	M/68
**2**	3.6	13	3	1	1	1	1	0	0	Cyclosporine, PUVA	Hypothyroidism, insulin resistance	M/43
**3**	5.1	10	6	1	1	2	1	1	1	Topical treatment	Diabetes, hypothyroidism	M/51
**4**	7.2	4	20	0	0	1	3	0	0	Topical treatment	-	F/36
**5**	8.2	8	12	1	1	1	3	1	0	Topical treatment	-	F/27
**6**	9.6	21	19	1	1	2	2	0	0	Topical treatment	Diabetes, hypothyroidism, neurosis nonalcoholic steatohepatitis obesity	F/61
**7**	13.2	13	16	1	1	1	2	1	0	Topical treatment	-	F/35
**8**	1.8	4	8	0	0	1	0	0	0	-	-	M/26
**9**	9.6	24	12	1	1	1	3	1	1	Methotrexate	Chronic obstructive pulmonary disease, hypertension	M/60
**10**	23.4	46	13	1	1	1	2	0	0	-	-	M/25
**11**	9.3	7	20	0	1	1	3	1	1	-	-	F/25
**12**	5	5	4	0	0	1	1	0	0	-	Dermatitis herpetiformis, diabetes	F/40
**13**	6.1	4	14	1	1	2	3	1	0	-	Coronary disease, diabetes	M/64
**14**	4.4	5	3	1	1	1	2	1	0	-	Hypertension	F/65
**15**	3.8	7	4	1	0	1	3	1	0	-	-	M/26
**16**	10.3	15	5	1	1	1	1	0	0	-	-	M/52
**17**	4.3	5	9	0	0	1	3	0	0	-	Epilepsy	F/29
**18**	7.6	19	4	1	1	1	1	0	0	-	Glaucoma, hypothyroidism	F/81

**Table 2:** Quantitation of the lysosomal-associated membrane protein 1 (LAMP1) marker in PP—psoriatic plaque and PN—psoriatic normal skin (in patients 1 to 7) in reference to clinical data including the PASI (Psoriasis Area and Severity Index), BSA (Body Surface Area), DLQI (Dermatology Life Quality Index), size of psoriatic plaque, thickness of psoriatic plaque, type of psoriasis, pruritus, arthralgia, psoriatic arthritis, gender, and age of each patient.

Patient No.	Quantification of LAMP1 in PP (Arbitrary Unit)	Quantification of LAMP1 in PN (Arbitrary Unit)	Ratio		
			PP/ PN	PP/ NN	PN/ NN
**6**	56.94	20.39	2.79	5.36	1.92
**7**	35.33	10.36	3.41	3.33	0.98
**2**	30.45	16.72	1.82	2.87	1.57
**3**	29.17	11.28	2.59	2.75	1.06
**5**	16.17	15.88	1.02	1.52	1.50
**4**	12.63	10.60	1.19	1.19	1.00
**1**	12.49	4.97	2.51	1.18	0.47

**Table 3:** Real-time qRT-PCR verification of selected genes related to the microphthalmia family (MiT family) (Panel A: *MITF*, *TFE3*, *TFEB*, and *TFEC*)*;* lysosomal marker genes and those encoding factors that control the lysosomal biogenesis process (Panel B: *LAMP1*, *MCOLN1*, *MTORC1*, *PPP3CA*, and *PPP3CB*); genes encoding lysosomal enzymes (Panel C: *ASAH*, *GLA*, *GM2A*, *HPSE*, *HYAL4*, *PSAP*, *SMPD1*, and *SPHK1*); and selected markers for psoriasis (*IVL*, *S100A7*, and *S100A9*) in the human epithelial cell model of HaCaT cells with “psoriasis-like” inflammation. The relative mRNA expression levels were determined against *TBP* (TATA-Box Binding Protein) in “cytokine mix”-activated HaCaT cells cultured in medium containing Ca^2+^ ≤ 0.1 mM vs. the non-activated HaCaT cell control cultured in medium containing either ≤0.1 or 2 mM Ca^2+^, and the relative quantitation was calculated using 2^−ΔΔ*C*t^ (FC—fold change). Bold font signifies a change in the expression level (≥1.3 and ≤0.7, respectively). Data represent the mean values ± standard deviation (SD) from *n* = 3; the non-activated HaCaT cell control cultured in medium containing a Ca^2+^ concentration ≤0.1; “cytokine mix”-activated HaCaT cells cultured in medium containing 2 mM Ca^2+^; and the non-activated HaCaT cell control cultured in medium containing 2 mM Ca^2+^.

Type of Group	Gene	*FC*	*SD*	*FC*	*SD*
		“Cytokine Mix”-Activated HaCaT at Ca^2+^ ≤ 0.1 mM vs. Non-Activated HaCaT Control at Ca^2+^ ≤ 0.1 mM		“Cytokine Mix”-Activated HaCaT at Ca^2+^ ≤ 0.1 mM vs. Non-Activated HaCaT Control at 2 mM Ca^2+^	
**A**					
MiT family transcription factors	*MITF*	0.83	±0.13	**0.67**	**±0.01**
	*TFE3*	1.06	±0.05	1.02	±0.05
	*TFEB*	0.87	±0.02	**0.74**	**±0.02**
	*TFEC*	0.91	±0.02	**0.71**	**±0.01**
**B**					
Lysosomal marker and genes encoding factors that control the lysosomal biogenesis process	*LAMP1*	1.05	±0.05	**0.19**	**±0.01**
	*MCOLN1*	0.83	±0.02	**0.43**	**±0.01**
	*MTORC1*	0.82	±0.05	**4.03**	**±0.25**
	*PPP3CA*	0.98	±0.04	1.17	±0.05
	*PPP3CB*	0.96	±0.03	1.05	±0.03
**C**					
Lysosomal enzymes	*ASAH*	0.83	±0.02	**0.6**	**±0.02**
	*GLA*	1.11	±0.07	1.12	±0.07
	*GM2A*	1.12	±0.07	**69.81**	**±4.69**
	*HPSE*	0.76	±0.04	0.76	±0.05
	*HYAL4*	**0.64**	**±0.14**	**0.01**	**±0.00**
	*PSAP*	**0.74**	**±0.09**	**0.58**	**±0.07**
	*SMPD1*	0.83	±0.02	0.95	±0.03
	*SPHK1*	**1.44**	**±0.14**	**0.65**	**±0.06**

Selected markers for psoriasis	*IVL*	**1.64**	**±0.98**	**0.32**	**±0.02**
	*S100A7*	**1.35**	**±0.06**	**1.68**	**±0.08**
	*S100A9*	**16.23**	**±0.48**	**45.27**	**±1.31**

## Supplementary Materials

Supplementary materials can be found at https://www.mdpi.com/1422-0067/20/9/2255/s1.
