# Correction: Bocheńska, K. et al. Lysosome Alterations in the Human Epithelial Cell Line HaCaT and Skin Specimens: Relevance to Psoriasis. *Int. J. Mol. Sci.* 2019, *20*, 2255

**DOI:** 10.3390/ijms21020594

**Published:** 2020-01-16

**Authors:** Katarzyna Bocheńska, Marta Moskot, Marcelina Malinowska, Joanna Jakóbkiewicz-Banecka, Aneta Szczerkowska-Dobosz, Dorota Purzycka-Bohdan, Joanna Pleńkowska, Bartosz Słomiński, Magdalena Gabig-Cimińska

**Affiliations:** 1 Department of Medical Biology and Genetics, University of Gdańsk, Wita Stwosza 59, 80-308 Gdańsk, Poland; katarzyna.bochenska@phdstud.ug.edu.pl (K.B.); marta.moskot@biol.ug.edu.pl (M.M.); marcelina.malinowska@biol.ug.edu.pl (M.M.); joanna.jakobkiewicz-banecka@biol.ug.edu.pl (J.J.-B.); joanna.plenkowska@phdstud.ug.edu.pl (J.P.); 2 Institute of Biochemistry and Biophysics, Polish Academy of Sciences, Laboratory of Molecular Biology, Kładki 24, 80-822 Gdańsk, Poland; 3 Department of Dermatology, Venereology and Allergology, Medical University of Gdańsk, Mariana Smoluchowskiego 17, 80-214 Gdańsk, Poland; aneta.szczerkowska-dobosz@gumed.edu.pl (A.S.-D.); purzycka-bohdan@gumed.edu.pl (D.P.-B.); 4 Department of Immunology, Faculty of Medicine, Medical University of Gdańsk, Dębinki 1, 80-211 Gdańsk, Poland; bartosz.slominski@gumed.edu.pl

The authors wish to make the following corrections to this paper [1].

In the original Figure 1A, the same image was mistakenly selected for two panels illustrating the lysosome number in HaCaT cells grown in medium containing a low concentration of calcium, non-activated or “cytokine mix”-activated. 

Due to mislabeling, replace it with the correct Figure 1A (Figure 1):

The authors would like to apologize for any inconvenience caused to the readers by these changes. This change has no impact on the conclusions.

## Figures and Tables

**Figure 1:**
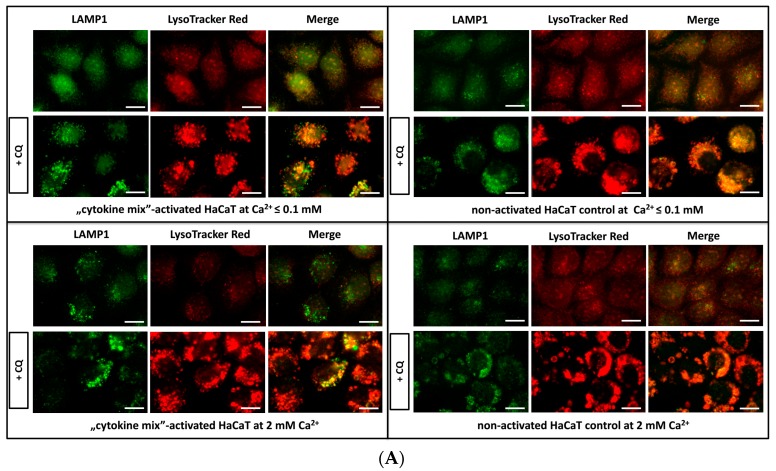
Fluorescence microscopy analysis of lysosomal compartments and other acidic vesicles in keratinocytes. Shown are representative microscopy images of HaCaT cells: (i) Activated with “cytokine mix”: interleukin 1 alpha (IL-1α), IL-17A, IL-22, oncostatin M (OSM), and tumor necrosis factor alpha (TNF-α) at a concentration of 2 ng/mL each constituent and cultured in medium containing Ca^2+^ ≤ 0.1 mM; (ii) non-activated HaCaT cell control cultured in medium containing Ca^2+^ ≤ 0.1 mM; (iii) “cytokine mix”-activated HaCaT cells cultured in medium containing 2 mM Ca^2+^; and (iv) non-activated HaCaT cell control cultured in medium containing 2 mM Ca^2+^. Control with chloroquine (CQ) was used to increase the total amount of lysosomes. All images were captured from randomly selected ten microscopic fields containing 50–100 cells, each from three independent experiments (*n* = 3), using a fluorescence microscope. Scale bar represents 0.25 µm. (**A**) LysoTracker Red DND-99 staining showing the distribution of acidic organelles, including mature lysosomes and lysosomal-associated membrane protein 1 (LAMP1) staining of the lysosomal membrane.
