# Supplementary material for: Lysosome Alterations in the Human Epithelial Cell Line HaCaT and Skin Specimens: Relevance to Psoriasis
Source: Int J Mol Sci. 2019 May 7;20(9):2255. doi: 10.3390/ijms20092255 (PMC6539968; doi:10.3390/ijms20092255)

Supplementary material:

Lysosome alterations in the human epithelial cell line HaCaT and skin specimens: relevance to psoriasis

Katarzyna Bocheńska[a], Marta Moskot[a,b], Marcelina Malinowska[a], Joanna Jakóbkiewicz-Banecka[a], Aneta Szczerkowska-Dobosz [c], Dorota Purzycka-Bohdan[c], Joanna Pleńkowska[a], Bartosz Słomiński[d], Magdalena Gabig-Cimińska[a,b*]

[a]*Department of Medical Biology and Genetics, University of Gdańsk, Wita Stwosza 59, 80-308 Gdańsk, Poland*
[b]*Institute of Biochemistry and Biophysics, Polish Academy of Sciences, Laboratory of Molecular Biology, Kładki 24, 80-822 Gdańsk, Poland*
[c]*Department of Dermatology, Venereology and Allergology, Medical University of Gdańsk, Mariana Smoluchowskiego 17, 80-214, Gdańsk, Poland*
[d]*Department of Molecular and Cellular Biology, Intercollegiate Faculty of Biotechnology UG-MUG, Abrahama 58, 80-307 Gdańsk, Poland*

[*]Corresponding author:
Tel. +48 58 523 6046, fax: +48 58 523 6025; E-mail: m.gabig@biol.ug.edu.pl

Table S1. Real-time qRT-PCR verification result of the microphthalmia family (MiT family) genes (*MITF, TFE3, TFEB* and *TFEC*), lysosomal marker genes and those encoding factors that control the lysosomal biogenesis process (*LAMP1, MCOLN1, MTORC1, PPP3CA* and *PPP3CB*), and genes encoding lysosomal enzymes (*ASAH, GLA, GM2A, HPSE, HYAL4, PSAP, SMPD1* and *SPHK1*) in skin specimens. The relative mRNA expression levels were determined against *GAPDH* (glyceraldehyde-3-phosphate dehydrogenase) mRNA level, and were calculated with $2^{-\Delta\Delta Ct}$ relative quantification and comparing between each group: (A) comparison between PP vs. NN, (B) PN vs. NN, and (C) comparison between PP vs. PN. Blue series – gene down-regulated (fold change, $FC \leq 0.7$); gray series – gene non-regulated ($1.3 > FC > 0.7$); red series – gene up-regulated ($FC \geq 1.3$). PP – psoriatic plaque, PN – psoriatic normal, NN – non-psoriatic normal, with n = 11 (psoriasis patients with numbers from 8 to 18) for PP/PN, and n = 11 (healthy individuals as a control with numbers from 5 to 15) for NN. Each experiment of real-time qRT-PCR analysis was repeated at least three times (technical repetition).

A

PATIENT NO.	*MITF*	*TFE3*	*TFEB*	*TFEC*	*LAMP1*	*MCOLN1*	*MTORC1*	*PPP3CA*	*PPP3CB*	*ASAH*	*GLA*	*GM2A*	*HPSE*	*HYAL4*	*PSAP*	*SMPD1*	*SPHK1*
8	0.15	0.00	0.11	0.02	0.34	0.34	0.23	0.01	0.02	0.00	0.04	1.51	1.46	0.66	0.03	0.00	0.03
9	0.12	0.02	0.04	0.00	0.57	0.27	0.22	0.03	0.01	0.00	0.16	3.26	3.88	2.83	0.04	0.00	0.04
10	1.37	0.16	0.19	0.01	2.40	4.38	4.88	0.09	0.06	0.06	9.62	332.12	5.77	6.38	0.87	0.02	0.73
11	0.27	0.04	0.05	0.04	4.33	6.03	46.06	0.12	0.06	0.15	25.03	88.37	112.81	0.02	1.27	0.18	2.49
12	0.73	0.11	0.18	0.03	9.03	13.76	7.29	0.13	0.10	0.33	25.73	18.97	91.00	99.27	2.75	0.13	3.69
13	0.19	0.01	0.03	0.01	3.02	8.83	19.37	0.31	0.03	0.15	16.06	20.19	106.73	251.31	0.67	0.07	47.67
14	0.07	0.01	0.04	0.04	1.43	2.30	1.22	0.80	0.03	0.12	5.12	2.61	29.61	67.81	1.39	0.01	19.09
15	0.14	0.01	0.04	0.01	2.32	4.97	2.65	0.25	0.04	0.09	11.52	36.90	69.44	163.52	1.09	0.06	1.41
16	0.12	0.04	0.03	0.01	8.25	18.15	10.82	0.09	0.05	0.14	18.45	158.19	81.45	138.46	1.20	0.20	2.12
17	0.07	0.03	0.04	0.02	2.47	3.54	4.19	0.07	0.03	0.05	9.42	83.03	55.63	0.01	0.94	0.11	1.27
18	0.10	0.13	0.05	0.01	7.70	22.82	29.77	0.06	0.05	0.34	38.47	9.48	103.09	0.10	2.77	0.00	4.68

B

PATIENT NO.	*MITF*	*TFE3*	*TFEB*	*TFEC*	*LAMP1*	*MCOLN1*	*MTORC1*	*PPP3CA*	*PPP3CB*	*ASAH*	*GLA*	*GM2A*	*HPSE*	*HYAL4*	*PSAP*	*SMPD1*	*SPHK1*
8	0.14	0.02	0.01	0.00	0.68	0.64	0.36	0.02	0.02	0.19	0.07	2.32	1.08	0.72	0.04	0.75	0.05
9	0.23	0.13	0.12	0.00	0.72	0.64	1.91	0.04	0.02	0.35	0.25	2.14	2.56	0.90	0.11	2.68	0.15
10	2.18	1.05	0.33	0.03	5.48	22.50	18.32	0.01	0.20	33.50	50.41	63.68	43.95	0.05	1.28	88.19	2.69
11	0.26	0.08	0.10	0.05	5.41	3.25	40.38	0.10	0.06	12.61	27.20	258.78	62.15	0.00	1.61	75.19	2.40
12	0.28	0.28	0.24	0.01	6.43	15.26	57.90	0.07	0.07	7.98	18.32	376.25	17.60	0.65	0.88	53.91	2.15
13	0.68	0.20	0.15	0.02	3.35	8.12	5.41	0.12	0.11	7.34	12.09	8.85	49.79	48.95	2.87	23.47	1.56
14	0.19	0.09	0.15	0.03	2.11	4.03	2.32	0.08	0.07	4.25	6.71	159.29	20.36	0.01	1.17	6.83	1.07
15	0.50	0.01	0.02	0.00	144.51	74.65	2.59	0.06	0.03	11.44	10.67	42.09	20.93	0.01	1.93	17.54	1.40
16	0.57	0.22	0.17	0.01	5.56	12.66	6.57	0.13	0.16	8.15	10.17	52.18	26.50	0.00	1.11	49.95	2.82
17	0.17	0.08	0.08	0.02	3.26	4.90	5.52	0.06	0.05	4.22	8.03	19.50	42.75	0.00	1.32	36.06	1.30
18	0.60	0.17	0.18	0.01	7.29	10.28	70.79	0.10	0.15	23.85	39.55	30.48	43.05	0.02	2.07	129.11	4.52

C

PATIENT NO.	*MITF*	*TFE3*	*TFEB*	*TFEC*	*LAMP1*	*MCOLN1*	*MTORC1*	*PPP3CA*	*PPP3CB*	*ASAH*	*GLA*	*GM2A*	*HPSE*	*HYAL4*	*PSAP*	*SMPD1*	*SPHK1*
8	1.04	0.07	0.25	0.68	0.50	0.53	0.24	0.40	0.71	0.42	0.60	0.65	1.36	0.91	0.57	0.90	0.58
9	0.53	0.13	0.34	1.43	0.80	0.42	0.11	0.84	0.33	0.45	0.62	1.53	1.52	3.14	0.41	0.36	0.26
10	0.63	0.15	0.56	0.27	0.44	0.19	0.75	8.63	0.30	0.11	0.19	0.12	0.13	0.00	0.68	0.10	0.27
11	1.04	0.55	0.49	1.13	0.80	1.85	1.14	1.20	0.95	0.69	0.92	0.03	1.82	7.06	0.78	0.87	1.04
12	0.14	1.51	1.21	0.72	4.17	2.27	0.25	0.89	0.82	2.07	0.48	0.01	1.27	4904.87	2.77	1.73	0.06
13	0.75	0.04	0.00	0.55	0.90	1.09	3.58	2.72	0.29	1.16	1.33	2.28	2.14	5.13	0.50	1.10	30.48
14	0.40	0.07	0.00	1.48	0.68	0.57	0.52	10.23	0.47	1.61	0.76	0.02	1.45	11993.79	1.19	0.74	17.88
15	0.27	1.02	1.91	2.69	0.02	0.07	1.02	4.07	1.39	0.48	1.08	0.88	3.32	3007.12	0.56	1.14	1.01
16	0.21	0.16	0.17	0.95	1.48	1.43	1.65	0.73	0.30	1.04	1.82	3.03	3.07	6553.01	1.08	1.43	0.75
17	0.38	0.31	0.44	1.07	0.76	0.72	0.76	1.11	0.66	0.62	1.17	4.26	1.30	21.56	0.71	1.11	0.98
18	0.16	0.33	0.26	0.86	1.06	2.22	0.42	0.57	0.34	0.83	0.97	0.00	2.39	7865.31	1.34	1.01	1.04

Figure S1. Percentage distribution of the expression of the microphthalmia family (MiT family) genes (*MITF*, *TFE3*, *TFEB* and *TFEC*), lysosomal marker genes and those encoding factors that control the lysosomal biogenesis process (*LAMP1*, *MCOLN1*, *MTORC1*, *PPP3CA* and *PPP3CB*), and genes encoding lysosomal enzymes (*ASAH*, *GLA*, *GM2A*, *HPSE*, *HYAL4*, *PSAP*, *SMPD1* and *SPHK1*) in skin specimens. Comparisons between PP vs. NN (right column), PN vs. NN (middle column), PP vs. PN groups (left column). Blue series – gene down-regulated (fold change, FC ≤ 0.7); gray series – gene non-regulated (1.3 > FC > 0.7); red series – gene up-regulated (FC ≥ 1.3). PP – psoriatic plaque, PN – psoriatic normal, NN – non-psoriatic normal, with n = 11 (psoriasis patients with numbers from 8 to 18) for PP/PN, and n = 11 (healthy individuals as a control with numbers from 5 to 15) for NN. Each experiment of real-time qRT-PCR analysis was repeated at least three times (technical repetition).

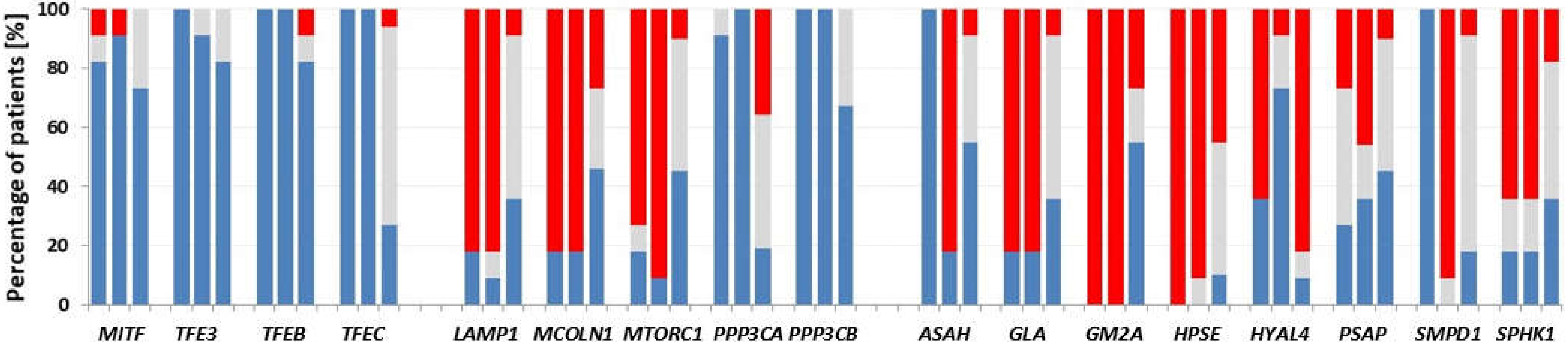

Supplement: Supplementary file 1 [file ijms-20-02255-s001.pdf]
